# Insights into the Coupling of Duplication Events and Macroevolution from an Age Profile of Animal Transmembrane Gene Families

**DOI:** 10.1371/journal.pcbi.0020102

**Published:** 2006-08-11

**Authors:** Guohui Ding, Jiuhong Kang, Qi Liu, Tieliu Shi, Gang Pei, Yixue Li

**Affiliations:** 1 Bioinformatics Center, Shanghai Institutes for Biological Sciences, Chinese Academy of Sciences, Shanghai, People's Republic of China; 2 Graduate School of the Chinese Academy of Sciences, Shanghai, People's Republic of China; 3 Institute of Biochemistry and Cell Biology, Chinese Academy of Sciences, Shanghai, People's Republic of China; 4 Shanghai Center for Bioinformation Technology, Shanghai, People's Republic of China; National Institute of Genetics, Japan

## Abstract

The evolution of new gene families subsequent to gene duplication may be coupled to the fluctuation of population and environment variables. Based upon that, we presented a systematic analysis of the animal transmembrane gene duplication events on a macroevolutionary scale by integrating the palaeontology repository. The age of duplication events was calculated by maximum likelihood method, and the age distribution was estimated by density histogram and normal kernel density estimation. We showed that the density of the duplicates displays a positive correlation with the estimates of maximum number of cell types of common ancestors, and the oxidation events played a key role in the major transitions of this density trace. Next, we focused on the Phanerozoic phase, during which more macroevolution data are available. The pulse mass extinction timepoints coincide with the local peaks of the age distribution, suggesting that the transmembrane gene duplicates fixed frequently when the environment changed dramatically. Moreover, a 61-million-year cycle is the most possible cycle in this phase by spectral analysis, which is consistent with the cycles recently detected in biodiversity. Our data thus elucidate a strong coupling of duplication events and macroevolution; furthermore, our method also provides a new way to address these questions.

## Synopsis

The interplay of information-processing life and force-driven environment has characterized Earth's evolutionary history since its beginning some 4 billion years ago. The study of macroevolution has seen a growing appreciation of this interplay. Previously, a large-scale effort was mounted to collect and analyze the paleontological and geochemical data. In the meantime, more and more genomes have been sequenced. The growing molecular sequence database with these paleontological data will provide important opportunities to investigate this interplay. Using the transmembrane proteins of 12 genomes, Ding and his colleagues have devised a sophisticated pipeline to date 1,651 duplication events grouped into 786 gene families, and have mapped the distribution of duplication events to the profile of macroevolution. They showed that the oxidation events played a key role in the major transitions of this density trace, and that the pulse mass extinction time points in the Phanerozoic phase coincide with the local peaks of the age distribution. Through some mathematical transformation of the density trace of the transmembrane gene duplicates during the Phanerozoic phase, they reported a potential cycle similar to the cycle detected by paleontologists. They concluded that a dramatically changed environment affected the evolution of life and left some imprint in the molecular level that can be detected.

## Introduction

Several models have been proposed to depict evolutionary trajectories of gene duplicates and evolutionary forces behind functional divergence of duplicate genes [1,2]. Despite the difference among detailed processes of the duplicates in these models, the final fate of the daughter copies is mostly determined by natural selection [2]. Therefore, drastic environmental alterations may result in frequent function fixations of duplicates. On the other hand, the environment variables play a crucial role in the genera-level evolution [3]. This concept compelled us to map the distribution of duplication events to the profile of macroevolution.

Previously, a large-scale effort was mounted to detect and analyze the cycles and patterns in macroevolution using paleontological and geochemical data [4–6]. These included paleontology methods such as finding patterns from the fossil records [7], geochemistry methods such as tracing the isotopic composition of the biogenic sediments [8], and ecological methods such as stochastic simulations of the ecosystem's environment-information transitions [9]. Studies on global marine fossil records [10] have obtained many interesting results, such as the relationship between macroevolutionary origination and CO_2_ levels [3], the phase shift between fluctuations in the rate of extinction and origination [11], and the mysterious 62-million-year (Myr) cycle, which has a high statistical significance but no physical or biological explanation [7]. Further studies [12,13] using sequence information to construct a gene's phylogenetic tree and compare it with the geological events inferred from other paleontological or geological studies implied that some speciation events were contemporaneous with the geological events. However, according to our knowledge, little research focused on the cycles and patterns in the gene duplication event records and the relationship between the evolutionary patterns on the molecular level and the species level. By using the animal transmembrane gene family, which is a key component for information exchange between cells and the environment and which can be easily investigated computationally [14], we detected the duplication events, estimated its age distribution with PAML (phylogenetic analysis by maximum likelihood) [15], and found that some patterns reported from macroevolution also emerged in the record of the duplication events.

## Results/Discussion

The age of the transmembrane gene duplicate was explicitly inferred as real time by the maximum likelihood method [16,17], which is a variable rate (so-called “relaxed clock”) method. Each orthologous group was supposed to have a smoothed rate, and multiple calibrations were used in each orthologous group to reduce the uncertainty in establishing a divergence time [18]. The S-clock (a clock inferred from synonymous distance) and rate-constancy clock were not used because the S-clock is highly unreliable for ancient gene duplications, while the assumption of rate constancy is often violated for distantly related genes [19]. Then, both the global clock and local clock models in the codeml program [16] of the PAML package [15] were applied to estimate the robustness of the different clock models used. The coefficient of Pearson correlation between time estimates by these two models in each divergence is 0.7439 (*p* < 2.2 × 10^−16^; Figure S2A), and the cumulative age distributions estimated by these two methods are almost the same (see Protocol S1 and Figure S2B), indicating that these two models are similar. The time estimates for further analysis were implemented by using the local clock model. In order to compare the patterns inferred from fossil records and the age distribution of transmembrane gene duplicates in the same time scale, we subdivided the age distribution of transmembrane gene families to the Phanerozoic and the Precambrian phases and focused on the Phanerozoic phase, which has been studied thoroughly in paleontology [5,6].

### Overall Age Distribution of Transmembrane Gene Families

**Figure 1:**
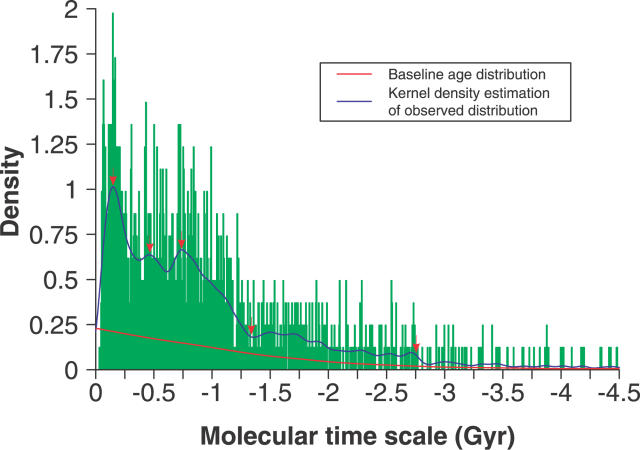
Density Histogram of Overall Transmembrane Gene Duplication Events The density trace (blue line) was obtained by using the Gaussian kernel density estimation with the bandwidth selected by Sheather and Jones' method [55]. The red line shows the estimate of the systemic bias in the overall distribution of duplication events, which was obtained by recalculating the mean value in each bin of 10,000 Monte Carlo–simulated histograms and smoothing the mean values with function “lowess” available in the statistics package of R environment [54]. The distinct transitions of the density trace are marked with red arrows.

The overall age distribution was determined on the basis of 1,620 transmembrane gene duplication events whose ages were estimated by the maximum likelihood method using the local clock model. Similar to the previous report [20], this distribution clearly shows three peaks (0.13 billion years [Gyr], 0.46 Gyr, and 0.75 Gyr ago approximately; Figure 1). It also reveals a phase when the density of the duplicates rose very rapidly (from 1.3 Gyr ago) and another phase with a slowly rising density (from 2.75 Gyr ago). In the distribution, the highest density of duplicates leans towards the youngest age classes, and the density drops off with increasing age. To assess the systemic bias of the age distribution, a simple Monte Carlo simulation was applied (see Materials and Methods). It seems that the systemic bias of the age distribution has little effect on the age distribution we have studied (*p* < 0.0001, nonparametric test). The observed age distribution deviates remarkably from the baseline distribution at about 2.75 Gyr ago (Figure 1). Thus, we ignored the bins whose age was before 2.75 Gyr for further analysis.

For the purpose of characterizing the feature of the actual age distributions, a null hypothesis was proposed. The null hypothesis is that the age distribution of the transmembrane gene duplicates is a uniform distribution, and assumes that the new transmembrane gene duplicates emerge, disappear, and fix in a continuous way with a constant evolutionary rate, a manner somewhat similar to the neutral theory in molecular evolution [21]. Then, the actual age distribution was tested against this null hypothesis by one sample Kolmogorov-Smirnov test. The null hypothesis was rejected and the age distribution was significantly uneven (D = 0.5318, *p* < 2.2 × 10^−16^, two-tailed Kolmogorov-Smirnov test). Consistent with the findings in human gene families [22], these findings support that both the “big-bang mode” and the “continuous mode” play significant roles in the transmembrane gene evolution.

#### Transmembrane gene duplication events versus multicellular complexity.

**Figure 2:**
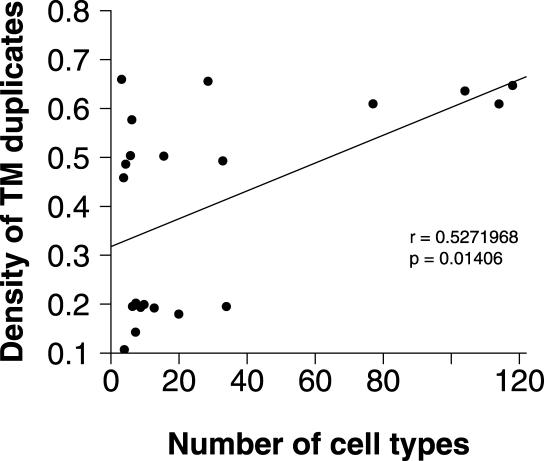
Correlation Analysis between the Maximum Number of Cell Types and the Density of the Transmembrane Gene Duplicates throughout the History of Life The estimates of the number of cell types in eukaryotes at different times in the past was derived from the work of Hedges et al. [23], and the corresponding density of duplicates was calculated by using linear interpolation in the time series inferred from the overall density trace (for details, see Protocol S1).

Considering that transmembrane proteins are the principal signal transduction mediators among cells, we investigated the correlation between multicellular complexity and the density of the membrane gene duplication events. The multicellular complexity, estimated by measuring the number of cell types and its corresponding time period, were cited from the work of Hedges et al. [23] (data shown in Table S2). For each time period in which the cell complexity was estimated, the density of duplicates was computed by linear interpolation in the time series inferred from kernel density estimation of overall distribution (see Protocol S1 and data shown in Table S2). As we expected, these two records display a positive correspondence (*r* = 0.5272, *p* = 0.01406, Pearson correlation analysis; Figure 2). This result indicates that at low multicellular complexity, duplication density may be either high or low, but at the four instances of high multicellular complexity, only high duplication density occurred.

#### Linkage with the oxidation events.

As illustrated by the arrows in Figure 1, some apparent transitions emerge from the age distribution. These observations raise an interesting question about the cause of this pattern during the transmembrane gene evolution. Since the oxygen levels in the environment and the ability of eukaryotes to extract energy from oxygen, as well as to produce oxygen, has been proposed as key factors in the rise of complex multicellular life [23], we next examined the relationship of the apparent disturbances of the age distribution with oxidation event records reconstructed from geochemical and fossil research, and the origin of some cell organelles related to the oxygen process deduced from molecular phylogeny analysis. We collected time periods of the origin of oxygenic photosynthesis, two great oxidation events (the origin of mitochondria and plastids), and the evolution of plants. Interestingly enough, the time point at which the density of the duplicates increases distinctly under the baseline distribution is completely consistent with the reliable minimum age [24] for the advent of oxygenic photosynthesis (2.75 Gyr ago) and the age of the domain Eucarya concluded from molecular fossils (about 2.7 Gyr ago) [8]. Moreover, the 0.74-Gyr peak is within the estimated time window for the second oxidation event, from 0.55 to 0.8 Gyr ago [12,25]; the 0.46-Gyr peak is within the time window for the early colonization of land by plants, from 0.48 Gyr to 0.36 Gyr ago [26]; the 0.13-Gyr peak is consistent with the age of the oldest flowering plant [27,28]. Especially, the origin of plastids (from 1.2 Gyr to 1.6 Gyr ago) [23,29] is nearly contemporaneous with the age (about 1.3 Gyr ago) when the density of the duplicates rose rapidly. In addition, the first oxidation event (from 2.0 Gyr to 2.4 Gyr ago) [4] and the origin of the mitochondria (from 1.5 Gyr to 2.3 Gyr ago) [23,30] coincide with the first phase in which the density of the duplicates rose slowly. The slowly rising density in this phase might have been caused by the global euxinic ocean, which kept deep-water anoxia beneath oxidized surface water about 1.84 Gyr ago [4,31].

These findings imply the linkage between the oxygen level and the transmembrane gene duplicates. For multicellular organisms, the oxygen level of the environment essentially determines the diffusion of oxygen across several layers of cells and the degree of communication among different cells. When the oxygen level rose, a potential multicellular niche might have given the transmembrane gene duplicates a chance to fix rather than experience loss or silencing. On the other hand, life is not a simple passive process, especially the ecosystem. The origin of oxygenic photosynthesis and the evolution of plants affected the earth's atmosphere and climate and increased the free energy supplied to the biota [32]. This factor provided a truly global environmental impact to animals, which can be detected by the correlation analysis of the age distribution of transmembrane gene duplication events with the time periods of geochemical events.

### The Record of Transmembrane Gene Duplication Events in the Phanerozoic Phase

**Figure 3:**
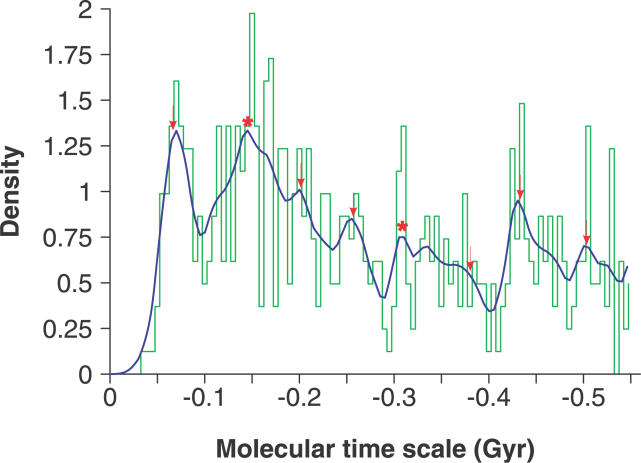
Density Trace of Transmembrane Gene Duplicates during the Phanerozoic Phase The trace was obtained by using the Gaussian kernel density estimation with a smaller “reasonable” bandwidth [33]. Arrows indicate the times of the major extinctions [5], and stars indicate the evolutionary events of plants [26–28].

Because most patterns of macroevolution were inferred from evidences in the Phanerozoic phase, and dating young duplication events is more reliable than ancient events using fossil calibration [22], we focused on the age distribution of the duplication events of transmembrane genes in the Phanerozoic phase. A smaller “reasonable” bandwidth [33] for the kernel density estimation (see Materials and Methods) was applied to extract some of the finer structures of the disturbances (Figure 3).

#### Transmembrane gene duplication events versus animal biological diversity events.

As expected from the logic relationship of oxygen levels and transmembrane gene duplication events, peaks of 0.13 Gyr and 0.32 Gyr appeared. The 0.13-Gyr peak is simultaneous with the time window of the spread of angiosperms mentioned above [27,28], while the rise from 0.3 Gyr to 0.4 Gyr coincides with the emergence of ancient forests implied from fossil density [26]. The evolved tree-like plant led to changes of most variables in the environment, such as the oxygen level, and this scenario fixed more transmembrane genes with neofunctions. Curiously, the other little distinct peaks corresponded well with the pulse extinctions (Figure 3) (i.e., the six great mass extinctions in the past 600 Myr [5]). This surprising finding indicates some relationship between the dramatic biological diversity decrease and the frequency of fixed transmembrane gene duplicates.

#### Cycles in the record.

**Figure 4:**
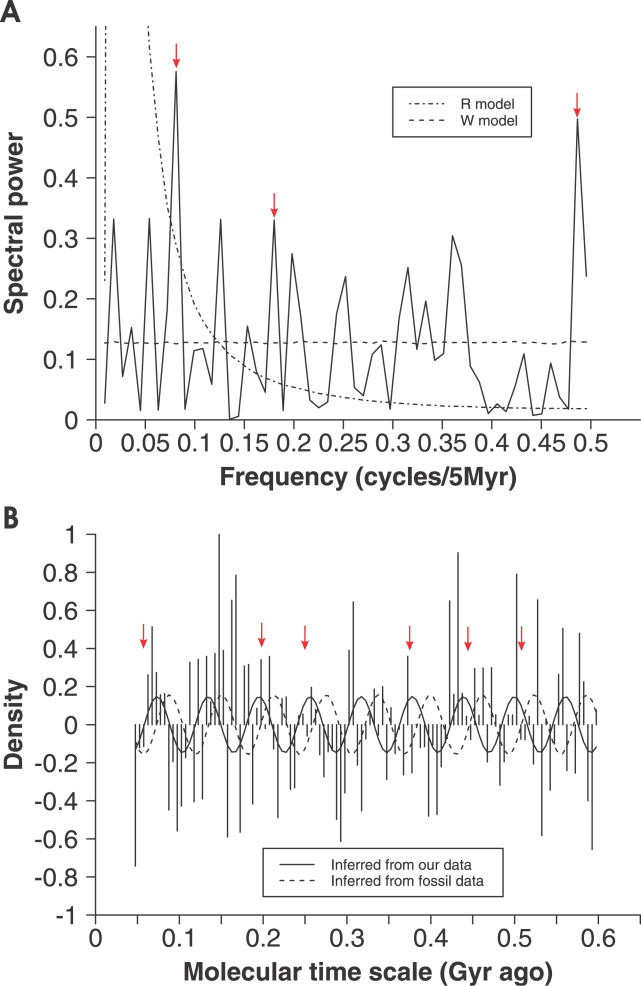
Periodogram Analysis of the Density Trace of Transmembrane Gene Duplicates during the Phanerozoic Phase (A) Fourier spectrum of the detrended density trace of the transmembrane gene duplicates during the Phanerozoic phase. Dot-dashed line (R model) and dashed line (modified W model) are estimates of spectral background [7]. The arrows indicate three potential peaks (Table S3), which are of statistical significance in finding the indicated peak at the specified period in either model, and accounts for at least 5% of the variance (i.e., biological significance cutoff) in the diversity signal. (B) The detrended density trace of the transmembrane gene duplicates during the Phanerozoic phase with a 62-Myr sine wave inferred from fossil data (solid line) [7] and a superimposed 61-Myr wave from our data (dashed line). The arrows indicate the times of major extinctions [5].

Because the periodogram is a more robust property to depict distributions, we performed decomposition in Fourier series of the detrended density trace of the duplicates in the Phanerozoic phase. Furthermore, we hypothesized that the same cycles, which have been detected in fossil record, would be found in the age distribution if macroevolution were coupling with duplication events. The density trace was detrended with a third-order polynomial curve, and then a periodogram was constructed with the detrended trace (Figure 4A). Modified models R and W [7] were applied in the Monte Carlo simulation with 10,000 repeats to determine the statistical significance (see Materials and Methods and Table S3). On this basis we defined a potential peak that has a statistical significance at the specified period in either model and incorporates at least 5% of the variance (i.e., biological significance cutoff) in the diversity signal [7]. We identified 3 potential peaks, which are 60.92-Myr, 27.29-Myr, and 10.32-Myr cycles. Because the 10.32-Myr cycle is only twice the interval of the data that are 5 Myr ago, we discarded this cycle value. The 60.92-Myr cycle has the strongest cyclicity in the density trace of the transmembrane gene duplicates (modified W model: *p* = 0.0098; R model: *p* = 0.14; representing 8.65% of the variance, the highest peak in the Fourier spectrum; Figure 4A). Consistent with the opinion that the macroevolutionary time series have characteristics of a random walk [34], because these cycles are not statistically significant, they cannot reject the null hypothesis of a random walk (Table S3).

The existence of the most potential cycle of 60.92 Myr in the age distribution of transmembrane gene families is a very interesting discovery, because it is not indistinguishable from the 62 ± 3-Myr cycle that is the most statistically significant cycle detected in biodiversity recently reported [7]. Furthermore, the 27.29-Myr cycle coincides with the 26- to 32-Myr cycle proposed by several reports [7]. Although 140 Myr cycles were previously reported [7], it was neither statistically nor biologically significant (i.e., accounting for less than 5% of the variance) in our data. We added the eliminated data in the first 45 Myr for the periodogram analysis and found no effect on the 61-Myr cycle, suggesting that the 61-Myr cycle in the data was robust, but the 27.29 Myr and 10.32 Myr cycles were not striking (unpublished data). These findings support our hypothesis mentioned above that the same cycles would be found both in fossil record and age distribution, and confirmed the emergence of the 62-Myr cycle from a completely independent dataset. Although the causes of the 62-Myr cycle in biodiversity remain mysterious, our data suggest that the variables that may be the geophysical and astronomical factors affecting the macroevolution also leave some imprint in the molecular level. Our data provide an evidence of this prediction.

We concentrated on the equation of the nonlinear least-squares fit of the 61-Myr sine wave to the residual data and compared it with the equation inferred from the fossil data [7], which can be represented as

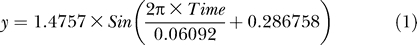
(our data) and

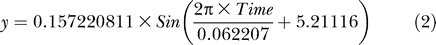
(fossil data). We noticed the significant difference between the phases of these two equations, which is 1.57π, indicating that the duplication events and the biodiversity are asynchronous. We found that all six major extinction events occurred during the expansionary phases of the density trace wave and correspond qualitatively with the declining phases of the 62-Myr cycle in biodiversity (Figure 4B).

### A Plausible Evolutionary Scenario

Our results seem to conflict with the intuition that the massive duplication events might go along with more speciation events or more biological diversity implicated in some previous works [35]. However, considering the variables triggering the mass extinction, a plausible evolutionary scenario could be proposed to explain these results. When the environment changed dramatically, the population of most species became smaller, or even extinct. Population genetic theory predicts that a change that may be deleterious to the gene function is ready to escape, purifying selection in a small population [21]. In addition, the sudden and various positive selections are beneficial for the fixation of the duplicates in most of the models depicting the gene duplicates' evolutionary trajectories [2]. Therefore, duplicates would be fixed in neofunction or subfunction, rather than purified or pseudogenized, when the stable biota was disturbed. Furthermore, genes coding transmembrane proteins mostly belong to the dosage-sensitive genes [36] that are beneficial for survival when redundant. Given that the paleoenvironment guided much of macroevolutionary development and showed a significant relationship with it [3], the frequency of the transmembrane gene duplication events was a significantly negative covariate with diversification for animals in the Phanerozoic phase. On the other hand, although our results are based only on the analysis of the transmembrane protein but not the whole proteomics, the findings from Arabidopsis thaliana and amphioxus gene families supported our results. The massive duplication events occurred in A. thaliana around 65 Myr ago [19], when Cretaceous-Tertiary extinction event took place and in Amphioxus about 488 Myr ago (mean value of the time phase from 300 to 680 Myr) [37]; that is at the Cambrian-Ordovician boundary, when many brachiopods and conodonts were eliminated and the number of trilobite species was severely reduced [38,39].

In this evolutionary scenario, it is not biological diversity but the environment variables that play a very important and basic role in the population dynamics of transmembrane gene duplicates. In this respect it is not surprising that some peaks in the age distribution are consistent with the oxygen level transition caused by the evolution of plants or others, even if these peaks did not couple with mass extinction. In addition, we defined mass extinction according to the fossils of animals. Thus, for animals, the increase of the oxygen level in the atmosphere would boost the animal evolution to more complex structures or diversity. However, oxygen would also make conditions “harsher” to certain organisms, such as anaerobic bacteria, which had little fossil record, and small increases in oxygen above 21% of the atmosphere increased the fire probability for forests [40]. Although the oxygen level transition did not trigger specific extinction in animals, these oxidation events corresponded with the dramatically changed environment, affecting the evolution of life. Our data show that the oxygen level in the atmosphere is the key variable determining the overall trends of age distribution of transmembrane gene duplicates.

### Conclusions

The data presented here clearly show that the duplication events of transmembrane genes are coupled with the macroevolution measurement and asynchronous with the animal biodiversity. The evolution history is a coevolution process of the environment and life [32,40] (e.g., the plant evolution versus oxygen level versus animal evolution). The overall shape of the age distribution is driven by the oxygen level in the atmosphere, while the waves of the distribution might be driven by some rhythmic external force. Furthermore, we proposed a plausible evolutionary scenario to explain these findings based on the factors finally determining the fate of the duplicates [1,2], which implies that the environment alternation would induce the redundancy of the existent genome system that is beneficial for survival in a rigorous condition. However, this system was not an optimized one and would resolve to different species, such as divergent resolution [41], when the environment disappeared.

In addition, we presented a methodology to provide a unique, temporally detailed understanding of the interaction of the transmembrane gene duplication events and the environment variables. Since the sequence data are thoroughly independent from the fossil record and more readily attainable, this methodology may give us a new strategy to validate patterns such as the 62-Myr cycle, which was detected from fossil or other geophysical records. Further studies using this method may offer important insights into the interplay of the microevolution and macroevolution factors.

## Materials and Methods

### Dataset.

Sequences of proteomes from 12 eukaryotes were obtained from the genomes section of National Center for Biotechnology Information (NCBI) ftp site (ftp://ftp.ncbi.nih.gov/genomes; accessed October 5, 2004). The species we chose are *Homo sapiens, Mus musculus, Rattus norvegicus, Gallus gallus, Drosophila melanogaster, Caenorhabditis elegans, Apis mellifera, Encephalitozoon cuniculi, Plasmodium falciparum, A. thaliana, Saccharomyces cerevisiae,* and *Schizosaccharomyces pombe.* ConPred II [42], a consensus approach combining the results of several proposed methods, was used to predict the secondary structure of the transmembrane protein after removing the signal peptide. Proteins with at least one transmembrane helix were regarded as transmembrane proteins. We identified 73,932 transmembrane proteins from the eukaryote proteomes chosen.

### Family building.

We developed a sophisticated pipeline to build the transmembrane gene families. This pipeline integrated the previously outlined strategy of COG [43,44] and HOBACGEN [45], and included some additional steps used in phylogenetic analysis, such as a bootstrapping test [46]. The detailed procedure information is explained in Protocol S1. Note that the E value cutoff of 10^−5^ was chosen based on an empirical method (see Figure S1) [47], and the bootstrapping cutoff value was 50% [46]. Each of the candidate families should have at least a triangle of genome-specific best hits (BeTs) [44] and two mammal homologous proteins. After a manual check of each candidate family, we had 863 homology families of eukaryote transmembrane proteins with multiple alignments for the tree calculation.

### Estimation of molecular time scale.

The topology of the phylogenetic tree of the candidate family was constructed by the neighbor-joining method [48] with Poisson distance. We also searched for the bacteria homologs in bacteria proteomes downloaded from the genomes section of NCBI ftp site by BLASTP analysis [49] with the default setting. The fungi, plant, and bacteria homologs were assigned as an outgroup to root the phylogenetic tree. In the absence of outgroup homologs, the root was placed at the midpoint of the longest route connecting two homologs. To minimize the error rate, gene families whose topology of the subfamily's tree was sharply in conflict with the uncontested animal phylogeny were excluded, and 797 homologous families were finally omitted. We then used these rooted phylogenetic tree topologies and their corresponding alignments to estimate the ages of gene divergences with multiple calibrations implemented in the codeml program [16] of the PAML package [15] (version 3.14). The mtREV24 model [50] was applied as a model of amino acid substitution and the α parameter for the γ distribution of evolutionary rates was estimated from the data by the program itself. We adopted several calibrations inferred from both fossil data and molecular data [29,51], such as mouse–rat (41 Myr ago), primate–rodent (91 Myr ago), mammal–bird (310 Myr ago), vertebrate–fly (993 Myr ago), vertebrate–nematodes (1,177 Myr ago), and animal–plant–fungi (1576 Myr ago). We used the global clock and local clock methods, respectively, to compare different rate models among branch groups [17]. For the local clock, each cluster of an orthologous group was assigned to its own branch rate group. To test the congruence between the global clock method and the local clock method, we collected and compared the age estimate of all divergences by these two methods (see Protocol S1 and Figure S2).

### Duplication event detection.

We identified subfamilies in each gene family along with the corresponding duplication events in terms of the constructed phylogenetic tree. The acceptance criterion was that two subfamilies (orthologs) emerging from a duplication event had at least two outparalogs [52] in different species, whereas a duplication event found in two different species simultaneously was a low probability event. Thus, we detected 1,651 duplication events in the final dataset with 786 gene families (detailed information and data are available at http://www.biosino.org/papers/TMEvol). All of the identified duplication events were recorded with the corresponding ages inferred from local methods. Among the final dataset, 100% included mouse, 97% rat, 92% human, 60% chicken, 27% fly, 23% worm, 10% cress, and 12% fungi.

### Histogram and kernel density estimates of duplication event ages.

Only duplication events with an age estimate of less than 4.5 Gyr were considered for further analysis, and 31 duplication events were excluded. The cutoff value of 4.5 Gyr was defined according to the age of the earth, which is about 4–5 Gyr old [53]. We represented the age distribution of duplication events by a 2-D density histogram with bandwidth of 5 Myr (Figure 1; data shown in Table S1). To assess small disturbances on the distribution, we employed kernel density estimation to the data because we considered it to be a straightforward procedure compared to splines and wavelets. We constructed the density traces by using the kernel density estimation function “density” with Gaussian kernel available in the statistics package of R environment [54] (version 2.0.1). Although the smooth bandwidth for the estimation was selected by an automatic method used before [55], we adjusted the bandwidth [33] for dealing with the data of different time phases by the parameter “adjust” in the function. The “adjust” was set at 1 (i.e., no adjustment to the automatic bandwidth selector) for the data of the overall density traces (Figure 1), and at 0.15 for the Phanerozoic phase data (Figure 3).

### Spectral analysis.

For the transmembrane gene duplication events occurring in the Phanerozoic phase (0–600 Myr ago), we constructed an age distribution (with an interval size of 5 Myr) and eliminated the bins in the first 45 Myr (approximately after the mouse–rat divergence) [29] in which almost no duplication events were detected, removed the third-order polynomial trend, and conducted a periodogram analysis with Fourier power spectrum by using the packages of R environment (version 2.0.1; function “lm” in statistics package for trinomial regression, “avgp” in GeneTS package [56] for Fourier transformation; Figure 4A). This workflow was similar to the process reported previously [7].

### Monte Carlo simulations.

We performed a Monte Carlo simulation to ensure that there was no systemic bias in the overall distribution of duplication event age. A number of ages of gene divergence (1,620) were randomly selected from all the ages recorded in the gene families without replacement each cycle. We then analyzed the randomly selected 1,620 ages and applied our procedure in exactly the same manner as we did for the real data, to get a histogram with bandwidth of 5 Myr; this process was repeated 10,000 times. We recalculated the mean value in each bin of 10,000 simulated histograms and got an average histogram to estimate a baseline age distribution (Figure 1). Here, Euclidean distance was used to define the difference between a pair of distribution, which is calculated as the square root of the sum of squared density differences between the distribution at each of the bins (see Protocol S1) [57]. The distances between 10,000 histograms and the average histogram were found to derive a frequency distribution. The mean distance is 4.351 ± 0.006856 Myr^−1^ (mean ± SEM), which is much lower than the distance of 48.30 Myr^−1^ between the observed distribution and baseline distribution (*p* < 10^−26^, nonparametric test).

To assess the statistical significance of the periodogram analysis in the Phanerozoic-phase duplication events, Monte Carlo simulation was carried out by using two different models (R model and W model) proposed by Rohde and Muller [7]. Our R model was simply a construction of random walks by randomly rearranging the steps between bins in the existing data. Monte Carlo simulations (10,000) were detrended (third-order polynomial) and analyzed. Their average spectral power was computed (Figure 4A). The modified W model was constructed by randomly scrambling the bins' order. Monte Carlo simulations (10,000) were transformed into power spectra by Fourier transformation. Their spectral power was averaged (Figure 4A) as well.

### Online material.

The members, phylogeny tree of each family, dates of duplication events, routines coded by R or Java, and other additional information related to this paper are available at http://www.biosino.org/papers/TMEvol.

## Supporting Information

Figure S1 Overall Distribution of E Values (261 KB JPG)

Figure S2 Robustness of the Molecular Time Scale Estimation with Two-Clock Model (262 KB JPG)

Protocol S1 Detailed Protocols of Methods Used in the Text (71 KB DOC)

Table S1 Raw and Baseline Density of the Transmembrane Gene Duplicates in Each Bin of the Histogram (72 KB XLS)

Table S2 Number of Maximum Cell Types versus the Density of Transmembrane Gene Duplicates at Different Times in the Past (18 KB XLS)

Table S3 Original Data Inferred for Fourier Transformations (30 KB XLS)

Table S4 List of Members in the Final Dataset with 786 Gene Families (768 KB XLS)

Table S5 List of Divergence Times in the 786 Gene Families (83 KB XLS)

## Abbreviations

Gyr: billion years
Myr: million years
