# Supplementary material for: Insights into the Coupling of Duplication Events and Macroevolution from an Age Profile of Animal Transmembrane Gene Families
Source: PLoS Comput Biol. 2006 Aug 11;2(8):e102. doi: 10.1371/journal.pcbi.0020102 (PMC1534073; doi:10.1371/journal.pcbi.0020102)
Supplement: Supplementary file 1 [file pcbi.0020102.sd001.doc]

Supporting Protocol

**The procedure of the family building**

Briefly, the procedure was as follows. (*i*). Filtering the widespread, typically repetitive domains (Tatusov et al. 2003). The domain prediction was performed by using RPS-BLAST program (Marchler-Bauer et al. 2002) (version 2.2.10) and the PSSMs for respective domains from CDD collection (Marchler-Bauer et al. 2005) (ftp://ftp.ncbi.nih.gov/genomes; November 5, 2004). This step will reduce the number of the false homologous protein pairs. (*ii*). All-to-all protein sequence similarity search from the transmembrane protein data set using BLASTP (Altschul et al. 1997) (version 2.2.10) with default setting. Low complexity sequences were filtered with SEG (Wootton and Federhen 1996), and 10^-5^ was chosen as the E value cut-off based on the overall distribution of expectation values over the entire protein space (Yona et al. 1999) (Fig. S1). The overall distribution of E values shown can be regarded as the average distribution of expectation values for a ‘typical’ protein sequence as a query; the steep slope at high expectation values suggests a rapid growth in the number of sequences that are unrelated to the query sequence (Yona et al. 1999). (*iii*). Detection of symmetrical best hits (BeTs). Blast output was filtered in a way to remove High-scoring Segment Pairs (HSPs) not compatible with a global HSPs arrangement on the protein sequence (Perriere et al. 2000). For complete protein sequences, two sequences in a pair were identified as BeTs if the remaining HSPs covered more than 80% of the protein length and if their similarity was more than 50% (two amino acids were considered similar if their BLOSUM62 similarity score was positive) and if both of them were complete (Perriere et al. 2000). Note that we simply detected the fragment sequence by text mining from annotation of the sequence. (*iv*). Single-linkage algorithm (nearest-neighbor clustering) (Duda et al. 2001) to the BeTs. So far, we had gotten the primal transmembrane gene family. (*v*). Splitting the promiscuous family into smaller but more reliable families by snipping the cut-edge (cut-edge is an edge whose deletion disconnect the graph) (Bondy and Murty 1982) of the protein similarity network. The protein similarity pairs in a protein space could be described as a similarity graph. Every gene family was a connected set in the similarity graph and the density in a connected region implied the degree of the similarity or homologous. It was reasonable to infer that a gene is included in a family if it has at least two best hits in the family. Thus, the cut-edge linking two densely connected regions artificially bridged several families to a promiscuous family. (*vi*). Iterative multiple alignment. Multiple alignments for the primal families were done with the program CLUSTALW (Chenna et al. 2003) (version 1.83). The parameters of the program were set as default. To decrease the false positive in a family, bootstrapping test was performed using CLUSTALW with 500 replicates and default setting for the output of multiple alignments. If the bootstrap proportion of a branch was less than 50% (Berry and Gascuel 1996), the protein members of this branch would be split into two new families. Multiply alignment and bootstrapping test were repeated with these new families until there was no new family generated. Our data was treated with this cycle 11 times to convergence, i.e. there was no branch with a bootstrap proportion less than 50%. (*vii*). Detection of triangles of genome-specific BeTs (Tatusov et al. 1997) and mammal homologous proteins. Each of the candidate families should be sure to have as least a triangle of genome-specific BeTs and two mammal homologous proteins. (*viii*). Manual check of each candidate family. Now, we got 863 homology families of eukaryote’s transmembrane protein with multiple alignments for the tree calculation.

**Comparison of the local clock and global clock**

10088 gene divergences marked with time scale estimated by both local clock and global clock in the transmembrane gene families were detected. We excluded the divergence whose time scale was larger than 4.5 Gyr (Woodhead 1999) by either local clock or global clock and 9860 divergence points were kept. Linear regression was applied to validate the correlation between the global method and the local method (Fig. S2A). The function of the linear fit is $\text{Local}=0\text{.}\text{924917}\times\text{Global}+0\text{.}\text{096573}$ (slope, p < 1.31×10^-08^; intercept, p < 2.2×10^-16^) and coefficient of correlation is 0.7439441 (t = 111.8063, p < 2.2×10^-16^). We also used cumulative distribution to illustrate the similarity of these two models (Fig. S2B). The red curve (local clock) almost superposes the entire green curve (global clock) in the Fig. S2B. These results suggest that the molecular clock dating by these two models are consistent.

**Euclidean distance computation**

The Euclidean distance was used to define the difference between a pair of profile/distribution (Malins et al. 1997). If the two distributions have no difference, the Euclidean distance of them was 0; otherwise, more difference, larger distance. In this paper, the distances were calculated between the Monte Carlo simulated distributions and the baseline distribution that was averaged from the 10,000 simulations. In general, the distance between two distributions $\vec{x}$ and $\vec{y}$ which can be treated as two vectors in a Euclidean space **R**^n^ is given by

$d=\left| \vec{x}-\vec{y} \right|=\sqrt{\sum_{i=1}^{n} \left| x_{i}-y_{i} \right|^{2}}$,	**[1]**

where $\vec{x}$ = (*x_1_*, *x_2_*, …, *x_n_*), $\vec{y}$ = (*y_1_*, *y_2_*, …., *y_n_*) and *n =* 450 in this paper which represents 450 bins in the histogram. The ten thousand distances were calculated to derive a frequency distribution. The distance of 48.30 between the observed distribution and baseline distribution is the far larger than the mean distance (p << 10-26, non-parametric test) for random distribution that is 4.351±0.006856 (mean ±SEM), implying that the systemic bias of the age distribution has litter effect on the age distribution we have studied.

**Linear interpolation**

Linear interpolation approximates the function on the interval [x1, x2] by the linear joining points (x1, f(x1)) and (x2, f(x2)), that is the straight line:

$\frac{f(x)-f(x_{1})}{x-x_{1}}=\frac{f(x_{2})-f(x_{1})}{x_{2}-x_{1}}$.	**[2]**

Based on this function, each *f(x)* ($x\in\left[ x_{1},x_{2} \right]$) can be calculated by

$f(x)=\frac{f(x_{2})-f(x_{1})}{x_{2}-x_{1}}\times(x-x_{1})+f(x_{1})$.	**[3]**

So, if two datasets used to correlation analysis are not well paired, the linear interpolation can be applied to overcome this problem. We used the linear interpolation to the larger dataset for appropriate point in the smaller dataset (Table S2) as the larger dataset with dense points will generate a reliable result. For correlation analysis between the maximum number of cell types and the density of the transmembrane gene duplicates throughout the history of life, the smaller dataset was the estimates of the number of cell types in eukaryotes at different times (Table S2); whereas for comparison of the genus diversity in macroevolution and the density of the transmembrane gene duplicates during the Phanerozoic phase, the smaller dataset was the age distribution of the transmembrane duplicates during the Phanerozoic phase. In addition, independent variables (variable x in Equation 3) in these two cases are the same, which is the time scale. It paired the different data sets for correlation analysis. The linear interpolation is implemented by using the function ‘approxfun’ in the base package of R environment (R Development Core Team 2005).

Altschul SF, Madden TL, Schaffer AA, Zhang J, Zhang Z et al. (1997) Gapped BLAST and PSI-BLAST: a new generation of protein database search programs. Nucleic Acids Res 25(17): 3389-3402.

Berry V, Gascuel O (1996) On the Interpretation of Bootstrap Trees: Appropriate Threshold of Clade Selection and Induced Gain. Mol Biol Evol 13(7): 999-1011.

Bondy JA, Murty USR (1982) Graph Theory with Application. Graph Theory with Application. New York: Elsevier Science Publishing Co., Inc. pp. 27-30.

Chenna R, Sugawara H, Koike T, Lopez R, Gibson TJ et al. (2003) Multiple sequence alignment with the Clustal series of programs. Nucleic Acids Res 31(13): 3497-3500.

Duda RO, Hart PE, Stork DG (2001) Pattern Classification. Pattern Classification. Second Edition ed. New York: Jonh Wiley & Sons. Inc. pp. 552-557.

Malins D, Polissar N, Gunselman S (1997) Models of DNA structure achieve almost perfect discrimination between normal prostate, benign prostatic hyperplasia (BPH), and adenocarcinoma and have a high potential for predicting BPH and prostate燾ancer. Proc Natl Acad Sci USA 94(1): 259-264.

Marchler-Bauer A, Panchenko AR, Shoemaker BA, Thiessen PA, Geer LY et al. (2002) CDD: a database of conserved domain alignments with links to domain three-dimensional structure. Nucleic Acids Res 30(1): 281-283.

Marchler-Bauer A, Anderson JB, Cherukuri PF, DeWeese-Scott C, Geer LY et al. (2005) CDD: a Conserved Domain Database for protein classification. Nucleic Acids Res 33(Database issue): D192-196.

Perriere G, Duret L, Gouy M (2000) HOBACGEN: database system for comparative genomics in bacteria. Genome Res 10(3): 379-385.

R Development Core Team (2005) R: A language and environment for statistical computing. Vienna, Austria: R Foundation for Statistical Computing.

Tatusov RL, Koonin EV, Lipman DJ (1997) A genomic perspective on protein families. Science 278(5338): 631-637.

Tatusov RL, Fedorova ND, Jackson JD, Jacobs AR, Kiryutin B et al. (2003) The COG database: an updated version includes eukaryotes. BMC Bioinformatics 4(1): 41.

Woodhead JA (1999) Geology. Geology. Pasadena: Calif. Salem Press. pp. 159.

Wootton JC, Federhen S (1996) Analysis of compositionally biased regions in sequence databases. Methods Enzymol 266: 554-571.

Yona G, Linial N, Linial M (1999) ProtoMap: automatic classification of protein sequences, a hierarchy of protein families, and local maps of the protein space. Proteins 37(3): 360-378.
